# Anti-Diabetic Effects of Ethanol Extract from *Sanghuangporous vaninii* in High-Fat/Sucrose Diet and Streptozotocin-Induced Diabetic Mice by Modulating Gut Microbiota

**DOI:** 10.3390/foods11070974

**Published:** 2022-03-27

**Authors:** Zi-Rui Huang, Li-Yuan Zhao, Fu-Rong Zhu, Yun Liu, Jian-Yong Xiao, Zhi-Chao Chen, Xu-Cong Lv, Ying Huang, Bin Liu

**Affiliations:** 1 College of Food Science, Fujian Agriculture and Forestry University, Fuzhou 350002, China; inaccelworld@gmail.com (Z.-R.H.); lsfood89@163.com (L.-Y.Z.); liuyun12086@163.com (Y.L.); fjxjy19@sina.com (J.-Y.X.); czc202202@163.com (Z.-C.C.); 2 National Engineering Research Center of JUNCAO Technology, Fuzhou 350002, China; lyrazfr@163.com; 3 Institute of Food Science and Technology, College of Biological Science and Technology, Fuzhou University, Fuzhou 350108, China

**Keywords:** *Sanghuangporous vaninii*, anti-diabetic effect, hyperglycemia, hyperlipidemia, gut microbiota

## Abstract

Type 2 diabetes mellitus (T2DM) may lead to abnormally elevated blood glucose, lipid metabolism disorder, and low-grade inflammation. Besides, the development of T2DM is always accompanied by gut microbiota dysbiosis and metabolic dysfunction. In this study, the T2DM mice model was established by feeding a high-fat/sucrose diet combined with injecting a low dose of streptozotocin. Additionally, the effects of oral administration of ethanol extract from *Sanghuangporous vaninii* (SVE) on T2DM and its complications (including hypoglycemia, hyperlipidemia, inflammation, and gut microbiota dysbiosis) were investigated. The results showed SVE could improve body weight, glycolipid metabolism, and inflammation-related parameters. Besides, SVE intervention effectively ameliorated the diabetes-induced pancreas and jejunum injury. Furthermore, SVE intervention significantly increased the relative abundances of *Akkermansia*, *Dubosiella*, *Bacteroides*, and *Parabacteroides*, and decreased the levels of *Lactobacillus*, *Flavonifractor*, *Odoribacter*, and *Desulfovibrio* compared to the model group (LDA > 3.0, *p* < 0.05). Metabolic function prediction of the intestinal microbiota by PICRUSt revealed that glycerolipid metabolism, insulin signaling pathway, PI3K-Akt signaling pathway, and fatty acid degradation were enriched in the diabetic mice treated with SVE. Moreover, the integrative analysis indicated that the key intestinal microbial phylotypes in response to SVE intervention were strongly correlated with glucose and lipid metabolism-associated biochemical parameters. These findings demonstrated that SVE has the potential to alleviate T2DM and its complications by modulating the gut microbiota imbalance.

## 1. Introduction

Type 2 diabetes mellitus (T2DM) is known as a chronic metabolic disease, and has widely spread around the world. The complications induced by T2DM are major public health concerns that are characterized by hyperglycemia, hyperlipidemia, and inflammation. Insulin resistance or secretion defect is principally the pathophysiological basis, resulting in the abnormal increase of blood glucose [1]. Inflammation is a causative factor involving obesity, diabetes, and cardiovascular diseases [2]. Therefore, inflammation plays an significant role in islet impairment and T2DM development [3]. Most of the commercially available anti-diabetes drugs, such as glucosidase inhibitors, insulin secretagogues, insulin-sensitizing agents, DPP-4 inhibitors, and GLP-1 analogs, have different side effects for certain symptoms and reactions.

Recently, studies on the gut microbiome revealed convincing evidence that the modulation of specific gut microorganisms is a potential therapy for the prevention of T2DM [4,5,6]. The gastrointestinal tract is a mass of trillions of microorganisms. Undigestible carbohydrates are available to the specific gut microbes, thus synthesizing functional metabolites [7]. Recently, the correlations between microbes and the development of cardiovascular disease and metabolic syndromes (MetS) have been clearer. For example, an atherosclerotic plaque has been shown to host its microbiota dominated by *Proteobacteria* [8]. A higher prevalence of Erysipelotrichaceae and Lachnospiraceae was found associated with metabolic disorders, and the *Holdemania* and *Blautia* genera correlated with clinical indicators of an impaired lipid and glucose metabolism [9]. It has been reported that *Faecalibacterium prausnitzii* and *Bifidobacterium* could modulate lipid metabolism and glucose homeostasis, and are associated with cardiovascular disease [10]. Furthermore, lower *Desulfovibrio* abundance and higher Bacteroides were related to the anti-diabetic effects of *Lactobacillus* intervention [11]. In addition, it has been reported that bile acids, one of the metabolites of gut microbiota, can be used as bioactive molecules to regulate circadian rhythms [12]. In particular, evidence has emerged that the gut microbiome is associated with T2DM [13] and T2DM-related complications, such as hyperglycemia [14], hyperlipidemia [15], and inflammation [16]. It has been proved that gut microbiota affects the efficiency of extracting energy from the diet [17,18]. By altering host metabolism and immune phenotypes, gut microbiota has a significant effect on energy expenditure, nutrient absorption, and immune regulation [19]. However, the functional connections between T2DM and gut microbiota are less well understood because of the complexity of the gut microbial community.

Ethanol extracts from various edible fungi have been reported to be beneficial to glucose metabolism, lipid metabolism, and gut microbiota [20,21,22,23]. For instance, oral administration of ethanol extract of *Ganoderma lucidum* could improve hyperlipidemia and intestinal flora composition by reducing the relative abundance of *Phascolarctobacterium*, *Globicatella,* and *Asaccharobacter*, and increasing the relative abundance of *Alistipes*, *Defluviitalea,* and *Alloprevotella* [24]. Pan et al. found that ethanol extract of *Grifola frondosa* attenuated the lipid metabolism disorder in high-sucrose/fat diet-fed rats, and also modulated the gut microbiota composition by promoting *Intestinimonas*, *Butyricimonas,* and *Helicobacter,* whereas reducing the abundances of *Turicibacter*, *Clostridium XVIII,* and *Romboustia* [25]. Besides, poricoic acid A, which is a tetracyclic triterpenoid compound extracted from *Poria cocos*, was investigated to ameliorate microbial dysbiosis, as well as attenuated hypertension and renal fibrosis [26]. It was indicated that ethanol extract of mushrooms may contain functional compounds to modulate the gut microbiota components. Modulating the gut microbiota by probiotics or bio-active natural products, especially promoting the proliferation of intestinal probiotics and inhibiting the growth/metabolism of harmful bacteria, would be a potentially effective approach to prevent or treat T2DM.

Diabetic patients have growing expectations for natural products with anti-diabetic activity. Unlike other chemical drugs with side effects, dietary supplements of medicinal fungi are more acceptable and popular for humans, such as *Ganoderma lucidum*, *Grifola frondose*, *Tremella aurantia*, *Cordyceps sinensis*, “Sanghuang”, etc. The scientific binomial of “Sanghuang” was long named as *Phellinus linteus*, *Inonotus sanghuang*, etc., for many years. Recently, the new genus *Sanghuangporus* was proposed, and comprised 14 species which are generally specialized with their host tree species, mainly containing *S. sanghuang* (traditional Chinese medicine)*, S. baumii* (second largest market), and *S. vaninii* (largest market) [27]. In addition, *Sanghuangporus*, as a portion of traditional food and natural medicine, has been used for 2000 years in China [28]. Modern medical research illustrated that “Sanghuang” exhibits many biological activities, containing anti-tumor, anti-bacterial, anti-inflammation, antioxidant, hypoglycemic, and hypotensive effects [29]. However, most of the previous studies focused on the extraction process, identification of active polysaccharide components, and physiological efficacy of water extract of *Sanghuangporus*. For example, Ma et al. purified a new exopolysaccharide from *S. sanghuang* P0988 broth (named SHP-2). SHP-2 showed a more complex structure, larger molecule weight, different anti-aging properties, and exhibited higher antioxidant enzyme activities [30]. Afterwards, it was reported that *P. linteus* polysaccharide extract administration had prebiotic effects in a diabetic rat model, which was indicated by enhancing insulin resistance and glycogen storage [31]. *P. linteus* polysaccharide also enhances the growth of the short-chain fatty acids (SCFAs)-producing microbes and SCFAs content in cecum, and helped to maintain homeostasis of lipid/lipoprotein profiles for ameliorating diabetes-associated liver and kidney injury [31,32]. However, few studies have attempted to describe the potential effects of ethanol extract from *S. vaninii* (SVE) on the composition of the gut microbiota, and its association with T2DM. The objective of this study was to evaluate the effects of SVE on anti-diabetes, and its complications based on the HFHS diet and streptozocin-induced T2DM mouse model. This study would provide a foundation for further exploration of the pharmacological action of “Sanghuang”.

## 2. Materials and Methods

### 2.1. Materials and Reagents

*Sanghuangporous vaninii* fruit bodies were bred and acquired from Hangzhou Academy of Agricultural Sciences (Hangzhou, China). The samples were dried and ground into powder (40 mesh), and stored at 4 °C. Chemical reagents were purchased from Shanghai yuanye Bio-Technology Co., Ltd. (Shanghai, China).

### 2.2. Preparation and Analysis of SVE

The extraction of ethanol extract from *S. vaninii* (SVE) was modified from previously reported methods [33,34]. In detail, the *S. vaninii* powders were exposed to 70% (*v*/*v*) ethanol (30:1, *v*/*w*), and were ultrasound (45 kHz, 300 W) at 60 °C for 1 h, and the supernatant was collected. The residues were then resuspended with the same volume of 70% (*v*/*v*) ethanol at 60 °C for 1 h. Finally, the mixture of supernatant was filtered, concentrated, and freeze-dried to obtain the SVE for further study. The main chemical composition of SVE was identified by UPLC-QTOF/MS according to the previously reported protocols [35].

### 2.3. Animal Models and Experimental Design

Forty male ICR mice (SPF, 4 weeks age) were supplied by Wu Shi’s Experimental Center Laboratory (Fuzhou, China), and they were raised under a condition of 23 ± 2 °C, a humidity of 55 ± 5%, and 12 h light/dark cycle. After one week of acclimation with food and water ad libitum, eight mice fed a normal diet were assigned to the normal control (NC, *n* = 8) group, and the rest of the thirty-two mice were fed with a high-fat and high-sucrose (HFHS) diet (15% lard, 15% sucrose, 1% cholesterol, 10% yolk, 0.2% sodium deoxycholate, and 58.8% chow) [36]. After 4 weeks of diet intervention, the mice with HFHS diet were intraperitoneally injected streptozotocin (STZ, dissolved in 0.1 M citrate buffer, pH 4.5) at a dose of 45 mg/kg, whereas the NC group mice were injected with the same volume of citrate buffer for three times a week. The mice with a fasting blood glucose (FBG) value over 11.1 mmol/L were regarded as diabetic mice, and were divided into four groups, including diabetic control (DC, *n* = 8), metformin (MET, 100 mg/kg/d, *n* = 8) [37], low dose of SVE (SVE-L, 100 mg/kg/d, *n* = 8), and high dose of SVE (SVE-H, 300 mg/kg/d, *n* = 8) groups [38].

### 2.4. Sample Collection and Biochemical Parameters

The body weight (BW) and fasting blood glucose (FBG) were measured every 2 weeks. Before the execution, all mice were measured and calculated for oral glucose tolerance test (OGTT), and area under the curve (AUC). The mice fasted overnight, and the feces were collected and quickly frozen in liquid nitrogen and kept at −80 °C. All mice were sacrificed by cervical dislocation after 4 weeks of intervention. The blood samples were collected, and the serum was separated by centrifugation at 3000 rpm for 10 min. The pancreas and jejunum were dissected, cleaned with PBS, and flash-frozen in liquid nitrogen, and stored at −80 °C [39]. In addition, the serum glycated serum proteins (GSP), total cholesterol (TC), triglyceride (TG), high/low-density lipoprotein-cholesterol (HDL-c and LDL-c), total bile acid (TBA), and free fatty acid (FFA) levels were measured by assay kits (Jiancheng, Nanjing, China). Moreover, fasting insulin (FINS), glucagon-like peptide-1 (GLP-1), interleukin-6 (IL-6), interleukin-10 (IL-10), and tumor necrosis factor α (TNF-α) were detected according to the manufacturer’s protocols of the ELISA kits (Chundu, Wuhan, China). Homeostasis model assessment-β (HOMA-β), HOMA-insulin resistance index (HOMA-IRI), HOMA-insulin sensitivity index (HOMA-ISI), and quantitative insulin sensitivity check index (QUICKI) levels were calculated [34,36].

### 2.5. Histopathological Analysis

The pancreas and jejunum were fixed in 4% paraformaldehyde overnight followed by dehydration through a series of ethanol solutions, and embedded in paraffin and cut into sections (5 μm thickness). The fabricated liver sections were stained by hematoxylin and eosin (H&E), and then observed with an optical microscope (Olympus, Tokyo, Japan).

### 2.6. Analysis of Gut Microbiota

Genomic DNA was extracted from feces following the previous protocol described [40]. The V3−V4 region of the bacterial 16S rRNA gene was amplified using universal primers 338F and 806R. The 16S rRNA gene sequencing libraries of bacteria were produced by using a TruSeq DNA PCR-Free Sample Preparation Kit (Illumina, San Diego, CA, USA). The high-throughput sequencing was carried out on the Illumina NovaSeq PE250 platform, which was carried out by Novogene Co., Ltd. (Beijing, China).

Principal component analysis (PCA) analysis of gut microbiota at the genus level was conducted by the SIMCA-14.1 (UMETRICS, Sweden). PICRUSt 2.0 was used to predict the intestinal microbial functional features, based on the KEGG, KO database. Variation analyses of different experimental groups were conducted using a linear discriminant analysis (LDA) effect size (LEfSe) algorithm (threshold > 3) through the Huttenhower Lab Galaxy Server (http://huttenhower.sph.harvard.edu/lefse/, accessed on: 18 November 2021). The relationship between the relative abundance of gut microbiota and biochemical parameters was calculated by Spearman’s rank correlation analysis, and visualized by the “psych” and “pheatmap” packages of R software (ver. 4.1.0) with |r| > 0.6, FDR adjusted *p* < 0.01, respectively. Cytoscape software (ver. 3.9.0) was used to show the correlation as a network intuitively.

### 2.7. Statistical Analysis

All the experiments were repeated at least three times. The values were defined as the mean ± standard deviation (SD). The differences were calculated using one-way ANOVA using GraphPad Prism 8. The significance of differences was described as * *p* < 0.05, ** *p* < 0.01, and *** *p* < 0.001 compared with the DC group, or ^#^ *p* < 0.05, ^##^ *p <* 0.01, and ^###^ *p* < 0.001 compared with the NC group.

## 3. Results and Discussion

### 3.1. Compound Composition Analysis of SVE Based on UPLC-QTOF/MS

The 100 g of *S. vaninii* fruit body could produce 10.57 g of SVE. In addition, a total of 664 chemicals (ESI+: 438; ESI−: 226) were detected by using UPLC-QTOF/MS. The main bioactive chemicals involved the alkaloid (2); terpene (4); isoflavonoids (8); flavonoids (12); and phenols (22), such as selagine, naringenin, curdione, equol, genistein, prunetin, luteolin, naringenin, nobiletin, hydroquinone, ethyl gallate, 3,4-dihydroxybenzaldehyde, etc. (Table S1).

### 3.2. Effects of SVE on Bodyweight and Glucose Metabolism Parameters

**Figure 1:**
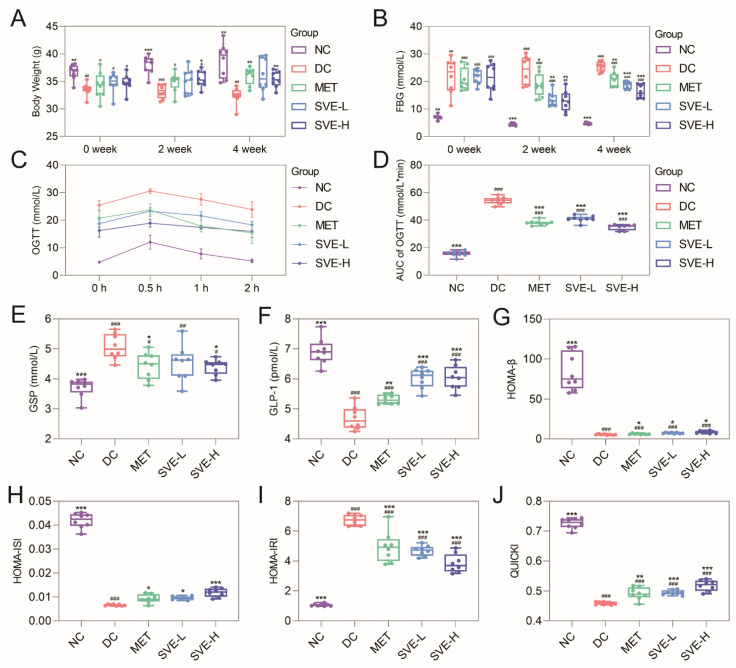
Effects of ethanol extract from *Sanghuangporous Vaninii* (SVE) intervention on the BW (**A**), FBG (**B**), OGTT (**C**), AUC of OGTT (**D**), GSP (**E**), GLP-1 (**F**), HOMA-β (**G**), HOMA-ISI (**H**), HOMA-IRI (**I**), QUICKI (**J**), in STZ-induced diabetic mice. Note: ^#^ *p* < 0.05, ^##^ *p* < 0.01, and ^###^ *p* < 0.001 vs. the NC group; * *p <* 0.05, ** *p* < 0.01, and *** *p* < 0.001 versus the DC group.

The HFHS diet could increase the BW of mice, and being overweight or obese increases the chances of having type 2 diabetes mellitus (T2DM) [41]. The cytotoxic agent, STZ, selectively destroys insulin homeostasis and integrity of β-cells of the pancreas to induce diabetes by entering through the Glut2 glucose transporter. Moreover, body weight is typically reduced in the STZ model of diabetes [42]. As shown in Figure 1A, compared with NC group mice, the HFHS diet and STZ injection induced a significant BW loss in DC (−8.47%, *p <* 0.01), MET (−6.65%, *p <* 0.05), SVE-L (−5.26%, *p* < 0.05), and SVE-H (−5.29%, *p <* 0.05) group mice at 0 weeks. With the development of the experimental period, the oral administration of MET strongly improved the BW of diabetic mice after 2 (+5.18%) and 4 (11.28%, *p <* 0.01) weeks. In addition, SVE-H significantly increased the BW compared to the DC group at 2 (+6.78%, *p <* 0.05), and 4 (9.21%, *p <* 0.01) weeks. However, the SVE-L group mice did not show significant BW changes compared to DC group mice. In short, the result indicated a dose-dependent effect of SVE on BW gain in diabetic mice. Similar results to ours have been reported in other studies, in which *Phellinus linteus* mycelia extract could effectively enhance the BW gain in diabetic mice [32]. Therefore, the utilization of SVE might increase BW through improving impaired energy metabolism, and enhancing carbohydrate utilization [42].

T2DM is characterized by disturbances in glucose and insulin metabolism, which results in hyperglycemia and insulin resistance [43]. Figure 1B illustrates the FBG of diabetic group mice were remarkably higher than NC group mice at 0 weeks (*p <* 0.01) and >11.0 mmol/L, which indicated that the model of T2DM was successfully established. SVE intervention could significantly decrease FBG levels compared to the DC group in the last 2 (*p <* 0.01) and 4 (*p <* 0.001) weeks. The OGTT is the clinical standard for diagnosis of T2DM [44], and GSP, a kind of protein that undergoes glycation and is in constant circulation in the blood, reflects the average blood glucose concentration in the past 1 to 3 weeks. GLP-1 receptor agonists (GLP-1 RA) are increasingly used in patients with T2DM. GLP-1 could improve insulin resistance in peripheral tissues to achieve a hypoglycemic effect [45]. Figure 1C-E shows that the SVE-H administration significantly reduced the diabetes-induced high AUC of OGTT (−35.66%, *p <* 0.001) and GSP (−13.31%, *p <* 0.05) levels. Simultaneously, SVE-L (+28.56%, *p <* 0.001) and SVE-H (+28.92%, *p <* 0.001) significantly enhanced the GLP-1 concentration in serum compared with the DC group (Figure 1F). In addition, MET, a positive control, had significantly reduced the FBG (18.32%, *p <* 0.01), AUC of OGTT (−29.75%, *p <* 0.001), and increased the GLP-1 (−12.56%, *p <* 0.05) levels in this study. In short, SVE could induce higher GLP-1 levels to improve hyperglycemia, and showed a dose-dependent effect.

Insulin resistance can reduce the intake of glucose, and lead to glucose accumulation, which leads to compensatory insulin production in islets [46]. Several surrogate indexes have been proposed based on fasting measurements of glucose and insulin for reflecting more accurately the T2DM. Compared to the NC group, the HOMA-β, used to evaluate basic insulin secretion, was significantly (*p <* 0.001) decreased in DC group, and was strongly reversed in SVE-L (*p <* 0.05) and SVE-H (*p <* 0.001) groups (Figure 1G). On the other hand, similar results had also occurred in the HOMA-ISI (Figure 1H), which is used to assess insulin sensitivity. HOMA-IRI and QUICKI were developed for evaluating insulin resistance [47]. In this study, both the SVE-L and SVE-H could significantly (*p* < 0.001) reverse the HFHS diet and STZ-induced high level of HOMA-IRI and low level of QUICKI. In conclusion, insulin secretion, sensitivity, and resistance were all improved after SVE intervention for 4 weeks, and these results are consistent with the previous finding [48].

### 3.3. Effects of SVE on Serum Lipid Parameters

**Figure 2:**
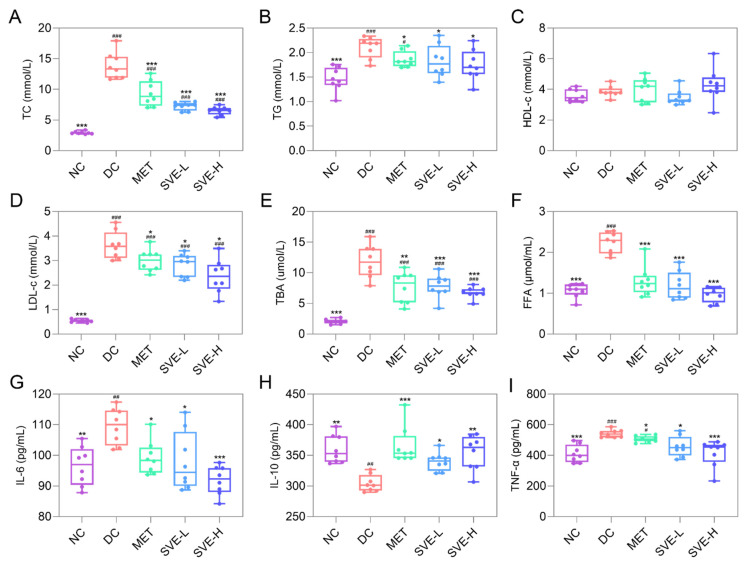
Effects of SVE intervention on the TC (**A**), TG (**B**), HDL-c (**C**), LDL-c (**D**), TBA (**E**), FFA (**F**), IL-6 (**G**), IL-10 (**H**), and TNF-α (**I**) in STZ-induced diabetic mice. Note: ^#^ *p* < 0.05, ^##^ *p* < 0.01, and ^###^ *p* < 0.001 versus the NC group; * *p* < 0.05, ** *p* < 0.01, and *** *p* < 0.001 versus the DC group.

T2DM can lead to multiple diabetes-related complications, which are responsible for the elevated death rates in T2DM. Thus, failure to detect and prevent T2DM in time may lead to serious complications, and pose a huge threat to life and health [49]. Usually, abnormal glucose metabolism and lipid metabolism often occur in parallel [50]. Afterward, consistent with previous studies [51,52], there were notable (*p <* 0.001) increases in the levels of TC (13.82 ± 2.18 mmol/L), TG (2.11 ± 0.22 mmol/L), LDL-c (3.62 ± 0.56 mmol/L), TBA (11.84 ± 2.69 μmol/L), and FFA (2.24 ± 0.26 μmol/mL) in the DC group compared to the NC group, whereas there were no meaningful differences regarding HDL-c levels among the groups (Figure 2A–F). After 4 weeks of treatment, the concentrations of TC, TG, LDL-c, TBA, and FFA decreased by 47.59%, 13.47%, 22.30%, 34.19%, and 46.94% in the SVE-L group, respectively, and decreased by 52.85%, 17.27%, 34.74%, 42.74%, and 56.49% in the SVE-H group, respectively. It was indicated that SVE may inhibit lipase activation, and decrease fatty acid mobilization from adipose tissue to improve the deficiency of insulin in the circulation system, and to ameliorate T2DM-induced lipid abnormalities (hyperlipidemia) [53]. Therefore, disorders of lipid metabolism are common in insulin-deficient diabetic patients.

### 3.4. Effects of SVE on Serum Levels of Inflammation Factors

Inflammation is the key trigger for the development of diabetes, and has been shown to contribute to increased diabetic complications [54]. Especially, IL-6 could impair glucose-responsive insulin secretion in islets. Additionally, insulin resistance in T2DM involves numerous pathophysiological effects, and TNF-α may play a key role in it [55]. Recent research has also suggested that IL-10 plays a regulatory role in diabetes [56], and IL-10 could suppress the induction and progression of autoimmune pathogenesis associated with diabetes to suppress the development of T2DM [57]. As shown in Figure 2G-I, the levels of IL-6 and TNF-α were significantly raised by 13.58% (*p <* 0.01) and 32.03% (*p <* 0.001), whereas the IL-10 level was significantly decreased by 15.50% (*p <* 0.01) in the DC group compared to the NC group. After 4 weeks of treatment, IL-6 was significantly reduced in the MET (−9.43%, *p <* 0.05), SVE-L (−10.55%, *p <* 0.05), and SVE-H (−16.18%, *p <* 0.001) groups, and TNF-α was also significantly decreased in the MET (−6.45%, *p <* 0.05), SVE-L (−15.63%, *p <* 0.05), and SVE-H (−23.71%, *p <* 0.001) groups. By contrast, IL-10 was elevated by 20.14%, 11.66%, and 16.46% in the MET (*p <* 0.001), SVE-L (*p <* 0.05), and SVE-H (*p <* 0.001) groups, respectively. IL-10 levels were found significantly elevated in SVE-L (*p <* 0.05) and SVE-H (*p <* 0.01) group mice. These results are in line with the findings in the previous study [58]. It was further suggested that inflammation might be an important pathogenetic factor in the development of insulin resistance in T2DM.

### 3.5. Effects of SVE on the Pathological Features of Pancreas and Jejunum

**Figure 3:**
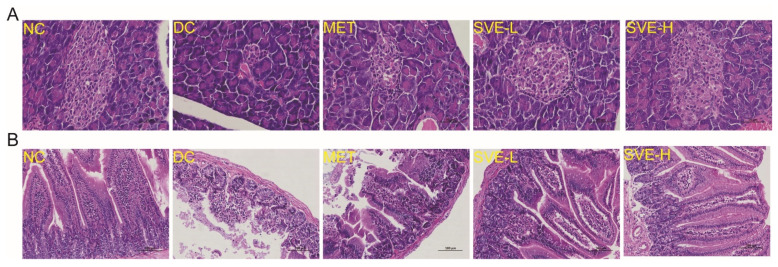
Histopathological analysis of pancreas (**A**) and jejunum (**B**) tissues in different groups with hematoxylin and eosin staining at 400× and 200× magnification, respectively. Note: The yellow letters in figures represent the different groups.

In addition, the pancreas is closely involved in the homeostasis of glucose, because it can not only secrete the glucose-lowering hormone, insulin, but also the glucose-promoting hormone, glucagon [59]. Figure 3A illustrated the structure of the pancreas was regular, and the islet cells in the NC group were arranged in a pattern without inflammatory cell infiltration. However, the islets in the DC group had some degeneration, and became smaller in size, the cells were polymorphic, and the cytoplasmic vacuolation was increased, β-cells were reduced, and the islets showed an atrophied appearance. Islet cells in the SVE-H group were morphologically normal and evenly distributed in the visual field. Thus, SVE intervention may reduce the shrinkage of pancreatic histiocytes, and restore the proportion of these cells somewhat, which is consistent with previous research [36].

On the other hand, a recent study reported that the proximal gut (small intestine) might regulate hepatic gluconeogenesis and muscle insulin resistance [60]. Moreover, it remains unclear where the jejunum fits in the development of diabetes. Figure 3B presented the histologic findings of the jejunum in different groups of mice. It was shown that the adventitia, muscular mucosa, and villi of jejunum tissues in the NC group were intact and clear. It was found that the HSHF diet and STZ-induced T2DM disrupted the villus structure (atrophic and thicker) in the jejunum to damage epithelial cells in the DC group. After treatment of SVE for 4 weeks, the number of villi was significantly increased and arranged with orderliness in the SVE-H group, whereas the submucosa of the SVE-L group was still thicker, which was similar to the MET group [61]. It was indicated that a high dose of SVE intervention plays a protective effect against T2DM-induced intestinal inflammation.

### 3.6. Effects of SVE on the Composition and Metabolic Function of Gut Microbiota

**Figure 4:**
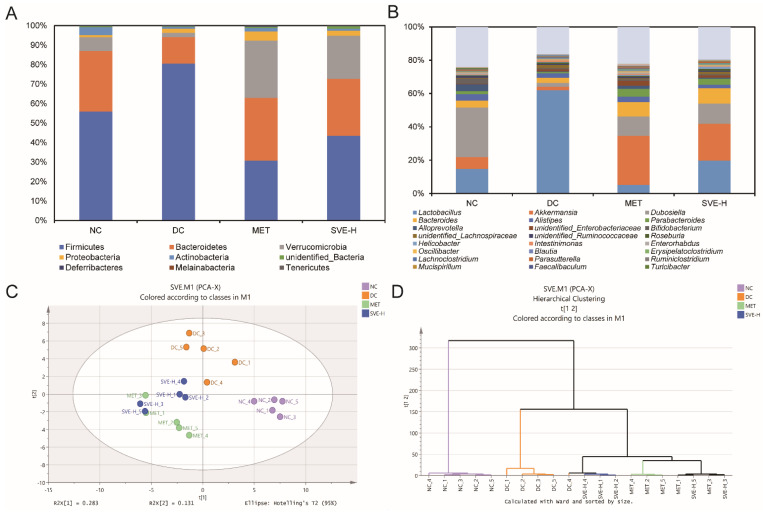
The components of gut microbiota at phylum (**A**) and genus (**B**) levels; PCA score (**C**) and hierarchical clustering (**D**) plots of gut microbiota at genus level in different groups.

The gut microbiota can directly or indirectly influence the host’s immune system, modulate inflammatory processes, and alter the expression of specific genes, thereby affecting the development of diabetes. Usually, gut microbiota profiles are not exactly comparable in diabetic patients [62]. In this study, based on the Illumina NovaSeq platform, the 16S rRNA gene was sequenced and analyzed to investigate the effects of SVE on the components of gut microbiota. At the phylum level (Figure 4A), Firmicutes, Bacteroidetes, Verrucomicrobia, Proteobacteria, and Actinobacteria played the dominant roles in the gut microbes of feces. Compared to the NC group, it was shown that the relative abundance of Firmicutes was strongly increased (+44.00%, *p <* 0.001), and the relative abundance of Bacteroides was decreased (−56.06%, *p <* 0.01) in the DC group, indicating that the value of Firmicutes/Bacteroides was significantly (*p <* 0.05) elevated in diabetic mice. These results were consistent with many previous findings on diabetic mice [63,64]. Additionally, MET and SVE intervention for 4 weeks had strongly reversed the gut components. The values of Firmicutes/Bacteroides were significantly decreased in the MET (*p <* 0.05) and SVE-H (*p <* 0.05) groups, which was reported to be correlated to the high FBG and inflammation status [65]. Several reports have found the increased value of Firmicutes/Bacteroides was associated with the development of diseases, including obesity, hyperlipidemia, hyperglycemia, and T2DM [66], indicating that SVE-H could modulate diabetes-induced gut dysbiosis.

At the genus level (Figure 4B), *Lactobacillus*, *Akkermansia*, *Dubosiella*, *Bacteroides*, and *Alistipes* were the most prominent genera. The relative abundances of *Lactobacillus* were 14.84%, 62.01%, 5.23%, 19.79%, and *Bacteroides* were 4.25%, 3.10%, 8.63%, 9.20% in the NC, DC, MET, and SVE-H groups, respectively. It was indicated that the relative abundances of *Lactobacillus* and *Bacteroides* were increased in the DC group compared with the NC group, whereas MET and SVE-H intervention for 4 weeks had partly reversed the alteration of gut microbiota induced by T2DM. By contrast, compared with the NC group, the HFHS diet and STZ injection strongly reduced the relative abundances of *Akkermansia* (NC: 7.12% DC: 2.14%, MET: 29.42%, SVE-H: 22.17%) and *Dubosiella* (NC: 29.58%, DC: 2.26%, MET: 11.59%, SVE-H: 12.08%) in the DC group, whereas that was elevated after MET or SVE-H treatment for 4 weeks. Interestingly, the *Alistipes* population was reduced in the DC group (2.77%), and increased in the MET group (3.18%), which showed a similar state to the NC group (3.86%). However, the SVE-H group (2.02%) showed a lower level of *Alistipes* than the DC group. The results also demonstrated SVE has the potential ability to regulate the composition of gut microbiota to achieve anti-diabetic effects.

Furthermore, PCA was used to compare the overall components of gut microbiota among these groups. The PCA score plot (Figure 4C) illustrated the significant separations among the NC, DC, MET, and SVE-H groups. Compared with the NC group, an obvious change along the negative direction of the first principal component (PC1) and the positive direction of PC2 in the DC group was demonstrated. Moreover, MET and SVE intervention explicitly changed PC2 along the negative direction due to T2DM, indicating that MET and SVE intervention could modulate the components of gut microbiota, and improve gut dysbiosis. However, MET and SVE also showed a shift along the negative direction of PC1. Besides, a hierarchical clustering plot (Figure 4D) demonstrated that the MET and SVE administration could shift the components of gut microbiota, and gut microbiota in these two groups were different from NC and DC groups, which was in correspondence with the PCA results. Briefly, SVE could play a similar role as MET to significantly modulate the components of gut microbiota in diabetic mice to ameliorate T2DM.

**Figure 5:**
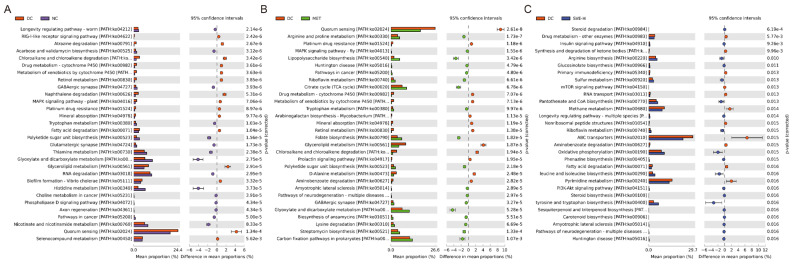
Function prediction of the gut microbiota revealed by PICRUSt2. DC versus NC (**A**), or MET (**B**), or SVE-H (**C**) group. Note: *p <* 0.05, confidence intervals = 95%.

PICRUSt2, an improved and customizable approach for microbial metabolic function prediction from 16S rRNA results [67], was conducted in this study. Based on the KEGG database, a total of 392 functional modules were mapped in NC, DC, MET, and SVE-H groups. Additionally, 203 functional modules were significantly changed between NC and DC groups (*p <* 0.05). Moreover, there were 15 up-regulated and 15 down-regulated modules at the top 30 modules (according to *p*-value) (Figure 5A), mainly containing thiamine metabolism (PATH:ko00730), glyoxylate and dicarboxylate metabolism (PATH:ko00630), glycerolipid metabolism (PATH:ko00561), RNA degradation (PATH:ko03018), nicotinate and nicotinamide metabolism (PATH:ko00760), quorum sensing (PATH:ko02024), selenocompound metabolism (PATH:ko00450), etc. There are 197 significant different expressed modules between DC and MET groups (*p <* 0.05) (Figure 5B), which were dominated by quorum sensing (PATH:ko02024), citrate cycle (TCA cycle) (PATH:ko00020), glycerolipid metabolism (PATH:ko00561), glyoxylate and dicarboxylate metabolism (PATH:ko00630), carbon fixation pathways in prokaryotes (PATH:ko00720), etc. Additionally, compared with the DC group, SVE-H intervention significantly altered the relative abundances of 179 functional modules (*p <* 0.05) (Figure 5C). Steroid degradation (PATH:ko00984), arginine biosynthesis (PATH:ko00220), methane metabolism (PATH:ko00680), ABC transporters (PATH:ko02010), oxidative phosphorylation (PATH:ko00190), and pyrimidine metabolism (PATH:ko00240) were the main different modules between these two groups. SVE-H administration significantly increased the relative abundances of insulin signaling pathway (PATH:ko04910), PI3K-Akt signaling pathway (PATH:ko04151), and IL-17 signaling pathway (PATH:ko04657) (*p <* 0.05), whereas it significantly decreased the relative abundances of fatty acid degradation (PATH:ko00071), glycerolipid metabolism (PATH:ko00561), and mTOR signaling pathway (PATH:ko04150) (*p <* 0.05). The modules are strongly associated with T2DM and T2DM-induced diseases [68,69]. These changes in metabolic pathways present insights into the underlying mechanisms of the alleviation of SVE on hyperglycemia, hyperlipidemia, and inflammation in mice induced by T2DM.

### 3.7. Correlations of the Key Gut Microbes with Biochemical Parameters

**Figure 6:**
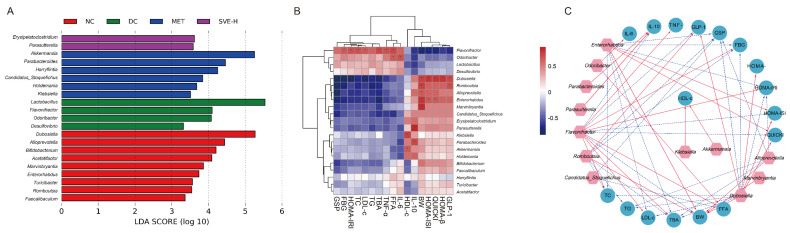
LEfSe analysis of gut microbiota at genus-level among NC, DC, MET, and SVE-H groups (**A**). Heatmap of correlations between the significant different gut microbiota and physiological/biochemical parameters (**B**). Visualization of the correlation network. Note: pink nodes: gut microbial genera; cyan nodes: parameters; red lines: Spearman’s rank correlation coefficient > 0.6, adjusted *p <* 0.01; blue lines: Spearman’s rank correlation coefficient <−0.6, adjusted *p <* 0.01; the linewidth indicates the strength of correlation (**C**).

The diversity of gut microbiota in different experimental groups was assessed based on LEfSe analysis. The histogram of the LDA score helped identify key microbes statistically, and explore the biomarkers in each group. Taxa with an LDA score threshold >3.0 were exhibited in the bar chart (Figure 6A). Dominant genera of 9, 4, 6, and 2 were discovered in the NC, DC, MET, and SVE-H groups, respectively. It was demonstrated that the NC group was characterized by higher relative abundances of *Dubosiella*, *Alloprevotella*, *Bifidobacterium*, etc. *Lactobacillus*, *Flavonifractor*, *Odoribacter,* etc. were the characteristic microbes in the DC group, indicating that gut dysbiosis occurred in T2DM mice; some studies also reported an increase in the above microbes resulting in high exposure to hyperglycemia, hyperlipidemia, and obesity [63,70]. The genera of *Akkermansia*, *Parabacteroides*, *Harryflintia,* etc. dominated the gut microbiota of the MET group, and at the same time, *Erysipelatoclostridium* and *Parabacteroides* played a predominant role in the SVE-H group. The result was consistent with many previous reports which regarded the *Akkermansia* spp. as a next-generation beneficial microbe to prevent diet-induced obesity, T2DM, and inflammation [71], indicating that the MET and SVE supplements could remarkably change the microbial features of diabetic mice.

Furthermore, Spearman’s correlation analysis was adopted to investigate the association between key microbes and biochemical parameters to evaluate the mechanism of SVE alleviating T2DM by regulating gut microbiota. The correlation heatmap (Figure 6B) showed that *Flavonifractor, Odoribacter*, *Lactobacillus*, and *Desulfovibrio* were positively related to the levels of GSP, FBG, HOMA-IRI, TG, TBA, TNF-α, FFA, and IL-6, whereas they were negatively associated with the levels of HDL-c, IL-10, BW, HOMA-ISI, QUICKI, HOMA-β, and GLP-1. By contrast, the rest of the key microbes showed an opposite relationship to the parameters. In particular, the squares marked with “*” were filtered by |r|> 0.6 and *p <* 0.01. In this study, oral administration of SVE may alleviate the glycolipid metabolism and inflammation-related biochemical parameters by modulating the specific gut microorganisms. Furthermore, all significant correlations of the heatmap were also visualized as a network plot in Figure 6C.

*Flavonifractor* was found to convert quercetin or other flavonoids into acetate and butyrate in the intestine [72]. However, *Flavonifractor* was reported to have a positive association with inflammation cytokines, such as interleukin, cholesterol, and LDL levels, in plasma [73]. Afterward, a higher abundance of pathogenic *Flavonifractor* was also detected in the DC group, and was strongly positively associated with the concentrations of FBG, HOMA-IRI, TC, LDL-c, TBA, TNF-α, and FFA, whereas this was significantly negatively related to the levels of IL-10, BW, HOMA-ISI, QUICKI, and GLP-1 in this study. This finding illustrated that increased *Flavonifractor* may lead to the occurrence of hyperlipidemia and hyperglycemia, increase oxidative stress, and trigger inflammatory markers. Similar results were also reported in some previous studies, in which the *Flavonifractor* population was elevated in multiple sclerosis patients and fa/fa model rats [74].

On the contrary, oral administration of SVE increased the specific microbial population, which was reported to have many anti-inflammatory and SCFA-producing abilities, such as *Bacteroides*, *Alloprevotella*, *Romboutsia*, *Akkermansia*, and *Bifidobacterium*. For example, *Dubosiella* showed a positive relationship to the levels of BW, HOMA-ISI, QUICKI, HOMA-β, and GLP-1, but was negatively associated with the GSP, FBG, HOMA-IRI, TC, LDL-c, TG, TBA, and TNF-α levels. It was reported that *Dubosiella* is closely associated with the development of many diseases, such as diabetes [75], abnormal lipid metabolism [76], and obesity [77], and has been used as a widely recognized probiotic in the therapeutic field [78]. The results indicated that SVE was proposed to influence the relative abundance of *Dubosiella* to ameliorate T2DM.

Besides, *Alloprevotella* belongs to short-chain fatty-acids-producing bacteria [79], and *Alloprevotella* plays a positive role in alleviating inflammation and non-alcoholic fatty liver. In the present study, the *Alloprevotella* population was reduced in mice of the DC group, and increases in the SVE-H group were significantly negatively correlated to levels of GSP, HOMA-IRI, TG, and TBA. Therefore, SVE administration could produce SCFAS to maintain the balance of the intestinal system to achieve the anti-diabetic effect eventually. On the other hand, *Romboutsia* could also produce SCFAs, and is linked to the degradation of L-fucose, glucose, and fructooligosaccharides [80]. The increase in *Romboutsia* was negatively associated with GSP, FBG, TC, LDL-c, TG, and TBA concentrations in present research, indicating that *Romboutsia* was positively related to the alleviation of T2DM and its complications [81].

Additionally, the *Enterorhabdus* population was significantly negatively associated with levels of GSP, FBG, HOMA-IRI, TC, LDL-c, TBA, TNF-α, and FFA levels, and positive associated with levels of BW, HOMA-ISI, and QUICKI. Previous findings also exhibited similar results to this study, in which the *Enterorhabdus* population was reduced in the hyperlipidemic group. Besides, the relative abundance of *Enterorhabdus* was negatively related to the hepatic and serum lipid parameters, and was also enriched in normal and experimental groups compared to the diabetic group mice [82]. However, in many other studies, the opposite results are reported as well. For example, Yang et.al found that the relative abundance of the *Enterorhabdus* was higher in prediabetic patients [83]. Another study illustrated that *Enterorhabdus* abundance was positively correlated with TC levels, which was also enriched in T2DM mice [84]. Hence, it was speculated that the 16S rRNA sequencing technology is limited in these studies, and combined metagenomics may be a more appropriate way to solve the controversy in further research.

Especially, *Akkermansia* in the intestine is a potential biomarker for the treatment of T2DM, and both *Akkermansia* and its phyla Verrucomicrobia were significantly decreased in this study, and SVE intervention strongly reversed this trend in this study. Furthermore, increased *Akkermansia* levels significantly elevated the concentration of HDL-c. This result was consistent with previous findings, in which *Akkermansia* population was found to be significantly decreased in high-fat diet-fed hamsters [85]. Recently, Lye et al. [86] have reported that *Lactobacillus fermentum* FTDC 8312 could increase the *Akkermansia* population and serum HDL-c levels in hypercholesterolemic mice at the same time. Coincidentally, dietary supplementation of black rice anthocyanin extract also increases the relative abundance of *Akkermansia*, which was reduced in C57BL/6J mice with hyperlipidemia and hypercholesterolemia [66]. This research demonstrated that *Akkermansia* could reduce fat accumulation, modulate lipid metabolism, and improve glucose–insulin homeostasis to improve the syndrome induced by T2DM.

## 4. Conclusions

This study revealed the hypoglycemic, hypolipidemic, and anti-inflammatory effects of SVE through a diabetic mouse model induced by a high-fat/high-sucrose diet combined with STZ. Moreover, SVE had a significant influence on the component and metabolic function of gut microbiota in diabetic mice. The relative abundances of *Dubosiella*, *Alloprevotella*, *Bifidobacterium*, *Lactobacillus*, *Flavonifractor*, *Akkermansia*, *Parabacteroides*, *Erysipelatoclostridium*, and *Parabacteroides* in diabetic mice were partly restored by SVE intervention. The gut microbes in response to SVE intervention were mainly enriched in the metabolic pathways, including glycerolipid metabolism, TCA cycle, steroid degradation, insulin signaling pathway, PI3K-Akt signaling pathway, IL-17 signaling pathway, fatty acid degradation, mTOR signaling pathway, etc. Additional work is required to investigate the in-depth mechanism of SVE administration on diabetes through fecal microbiota transplant and multi-omics (transcriptomics, proteomics, and metabolomics) technology. Overall, these discoveries deliver a scientific underlying for the further understanding of the anti-diabetic effects and application of SVE in ameliorating diabetes.

## Data Availability

Not applicable.

## Supplementary Materials

The following supporting information can be downloaded at: https://www.mdpi.com/article/10.3390/foods11070974/s1, Table S1: The main compounds of SVE using UPLC-QTOF-MS/MS.
